# Liquid Chromatography–High-Resolution Mass Spectrometry (LC-HRMS) Fingerprinting and Chemometrics for Coffee Classification and Authentication

**DOI:** 10.3390/molecules29010232

**Published:** 2023-12-31

**Authors:** Nerea Núñez, Javier Saurina, Oscar Núñez

**Affiliations:** 1 Department of Chemical Engineering and Analytical Chemistry, University of Barcelona, Martí i Franquès 1-11, E08028 Barcelona, Spain; xavi.saurina@ub.edu (J.S.); oscar.nunez@ub.edu (O.N.); 2 Research Institute in Food Nutrition and Food Safety, University of Barcelona, Recinte Torribera, Av. Prat de la Riba 171, Edifici de Recerca (Gaudí), Santa Coloma de Gramenet, E08921 Barcelona, Spain; 3 Serra Húnter Fellow, Departament de Recerca i Universitats, Generalitat de Catalunya, Via Laietana 2, E08003 Barcelona, Spain

**Keywords:** non-targeted LC-HRMS analysis, fingerprinting chemical descriptors, coffee adulteration, principal component analysis (PCA), partial least squares–discriminant analysis (PLS-DA), partial least squares (PLS) regression

## Abstract

Nowadays, the quality of natural products is an issue of great interest in our society due to the increase in adulteration cases in recent decades. Coffee, one of the most popular beverages worldwide, is a food product that is easily adulterated. To prevent fraudulent practices, it is necessary to develop feasible methodologies to authenticate and guarantee not only the coffee’s origin but also its variety, as well as its roasting degree. In the present study, a C18 reversed-phase liquid chromatography (LC) technique coupled to high-resolution mass spectrometry (HRMS) was applied to address the characterization and classification of Arabica and Robusta coffee samples from different production regions using chemometrics. The proposed non-targeted LC-HRMS method using electrospray ionization in negative mode was applied to the analysis of 306 coffee samples belonging to different groups depending on the variety (Arabica and Robusta), the growing region (e.g., Ethiopia, Colombia, Nicaragua, Indonesia, India, Uganda, Brazil, Cambodia and Vietnam), and the roasting degree. Analytes were recovered with hot water as the extracting solvent (coffee brewing). The data obtained were considered the source of potential descriptors to be exploited for the characterization and classification of the samples using principal component analysis (PCA) and partial least squares–discriminant analysis (PLS-DA). In addition, different adulteration cases, involving nearby production regions and different varieties, were evaluated by pairs (e.g., Vietnam Arabica—Vietnam Robusta, Vietnam Arabica—Cambodia and Vietnam Robusta—Cambodia). The coffee adulteration studies carried out with partial least squares (PLS) regression demonstrated the good capability of the proposed methodology to quantify adulterant levels down to 15%, accomplishing calibration and prediction errors below 2.7% and 11.6%, respectively.

## 1. Introduction

Recently, the analytical requirements of food trials have been augmented considerably because food fraud is a growing challenge worldwide and food safety is often difficult to control due to extensive commercial chains. In addition, consumer expectations about food quality have also increased, being willing to pay more money for safer food products with specific attributes, such as the geographical origin of production, among others. To cope with all these factors, the development of analytical methodologies to guarantee food integrity, safety and authenticity has become of paramount importance since it is a criterion of quality and safety for them [1,2]. These analytical methodologies mainly focus on targeted strategies based on monitoring selected compounds used as sample markers (chemical descriptors) to accomplish their classification, characterization and authentication [3]. Thus, if standards are commercially available and the methods are perfectly established, targeted strategies are very powerful and suitable for carrying out food quality control and authentication. However, quantifying a relatively high number of target compounds in complex matrices such as foodstuffs is difficult because of both possible matrix effects and/or interferences [3,4]. In addition, the requirement of standards, which are not always commercially available, will considerably increase the cost of such methodologies. All these facts are among the main drawbacks of targeted approaches. In this respect, non-targeted strategies, based on obtaining a sample metabolomic fingerprint, have gained in importance in the last few years to deal with a wide variety of analytical issues related to food authenticity, food safety and public health. In these strategies, prior knowledge of the chemical compounds that may be present in the samples is not necessary as the sample fingerprint consists only of the instrumental response of a given method [1,4,5,6,7]. For example, ultraviolet or fluorescence data as a function of chromatographic time [8], or the intensity of MS signals as a function of the *m*/*z* ratio and time [9] are examples of instrumental responses typically employed as sample fingerprints to solve food authentication issues. Thus, non-targeted methods can maximally explore the compounds present in the sample [1].

As commented, non-targeted methodologies have been widely employed to address different food issues, such as verifying geographical origin or detecting contaminants [1,10,11]. According to the literature, the most common analytical methods for food fingerprinting metabolomics rely on HRMS [6,12] and nuclear magnetic resonance (NMR), especially when a tentative identification of metabolites is also intended [2,5,6,7,12,13,14]. However, fingerprinting methodologies based on ultraviolet–visible (UV-vis) and fluorescence (FL) detection are also widely used to address food characterization and authentication [15]. Due to the food matrix complexity, separation techniques such as liquid chromatography (LC), gas chromatography (GC) or capillary electrophoresis (CE) are considered in combination with the mentioned detectors to increase analyte resolution [16,17,18]. As previously commented, general and simple sample treatment methods are frequently preferred to reduce chemical compound discrimination prior to analysis. Finally, fingerprinting metabolomic strategies provide, in general, a large quantity of data that, together with the high number of samples involved in food authentication issues, make necessary the use of chemometrics to extract useful information [4,6,7].

Beverages are easily alterable through fraudulent practices, such as mislabeling or the addition of unspecified additives to increase their volume, with fruit juices, coffee, tea, wine and other alcoholic beverages being the ones with the highest adulteration rates [19]. Coffee is the most important commercial non-alcoholic beverage, being the second most commercialized product in the world markets after petroleum, with a turnover of ca. USD 10,000 million per year [20]. The coffee intake brings healthy effects against cardiovascular diseases, obesity, hypertension, type II diabetes or stress due to the antioxidant activity of bioactive substances such as polyphenols [21]. Worldwide, the two principally cultivated coffee species are *Coffea arabica* (Arabica coffee) and *Coffea canephora* (Robusta coffee). In general, coffee production areas extend from 25 N to 25 S of latitude because of the appropriate climatic conditions for coffee cultivation. In addition, coffee beans produced at high altitude are harder to produce and, therefore, more appreciated. The sites for coffee growing are then selected based on environmental factors like temperature, sunshine intensity, wind, type of soil, topography, rainfall and humidity, among others. Depending on these factors, the content of bioactive substances in coffee varies, resulting in coffees with very different properties and flavors [22]. These production factors continue attracting the interest of coffee breeders, especially in the case of Arabica coffee which, in general, is the most preferred by consumers and is considered of higher quality than Robusta coffee. Apart from the species, the final coffee price also depends on the region of production and agricultural practices [20,22]. For these reasons, coffee is highly susceptible to fraudulent practices for illicit benefits. Thus, producers and importing companies are interested in analytical methods to guarantee that coffee has not been adulterated along the complex commercial chain [22].

To address coffee characterization and authentication, both targeted and non-targeted strategies have been proposed, the latter being the most employed in the last few years. NMR [23] and infrared (IR) [24] spectroscopies, high-performance liquid chromatography with ultraviolet (HPLC-UV) or fluorescence (HPLC-FLD) detection [11,15], direct infusion–electrospray–high-resolution mass spectrometry (DIESI-HRMS) [25], and gas chromatography coupled to mass spectrometry (GC-MS) [26,27] are examples of techniques for coffee authentication via non-targeted fingerprinting. Some LC-HRMS metabolomic approaches have also been described for the evaluation of the coffee roasting process [28] or the assessment of Colombian coffees [29].

In the present work, a liquid chromatography–high-resolution mass spectrometry (LC-HRMS) method using a linear ion-trap (LTQ)–Orbitrap mass analyzer was evaluated to address the classification, characterization and authentication of coffee beverages according to their origin, variety and roasting degree. Detection through HRMS was selected because it is capable of identifying unknown components and reducing false positives or negatives, making it crucial in obtaining comprehensive and reliable profiles in sample analysis. Additionally, it contributes to the identification of metabolites, providing valuable information for developing future targeted methods [28,29]. For this study’s purposes, a total of 306 commercially available coffee samples grouped into three sets were analyzed with the proposed methodology after simply brewing the coffee and filtering. The obtained LC-HRMS fingerprints based on the feature intensity as a function of the *m*/*z* ratio and chromatographic time were then used as the source of chemical information (sample chemical descriptors) to address the characterization and classification of the analyzed coffees by principal component analysis (PCA) and partial least squares-discriminant analysis (PLS-DA). Finally, some coffee adulteration cases were defined to evaluate the capability of the proposed methodology to detect and quantify coffee adulterations with partial least squares (PLS) regression to prevent future coffee fraud. Additionally, it must be highlighted that in this work, all the sample characteristics addressed (coffee origin, variety, and roasting degree) will equally contribute to the obtained sample profiles, thus addressing specific classification issues while considering different features simultaneously, in contrast to previous studies where certain sample features are fixed during the analysis. 

## 2. Results

### 2.1. Non-Targeted LC-HRMS Fingerprints

Nowadays, LC-HRMS is one of the most outstanding techniques in food analysis due to its high specificity, sensitivity and selectivity. In this work, the non-targeted LC-HRMS metabolomic fingerprints of coffee samples were obtained by reversed-phase chromatography using a porous-shell C18 column under gradient elution conditions (see Section 3.2) with acidified water (0.1% formic acid) and methanol as mobile phase components. The HPLC system was coupled to an LTQ Orbitrap Velos HRMS instrument (Thermo Scientific, Pleasanton, CA, USA) using an electrospray ionization source (ESI) in negative ion mode. 

**Figure 1:**
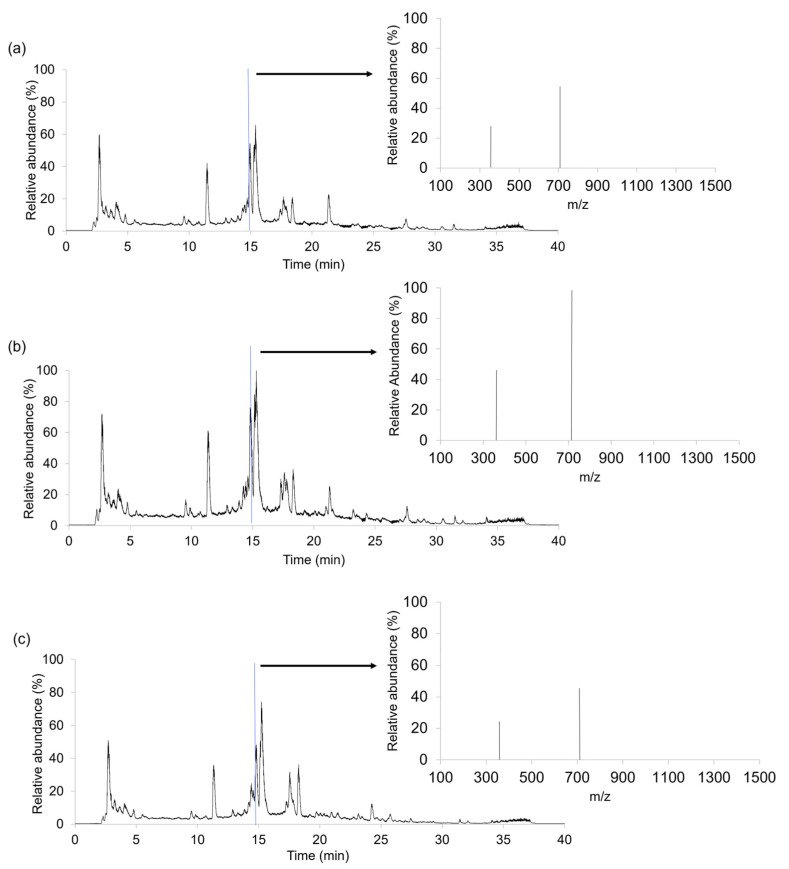
LC-HRMS metabolomic fingerprints (total ion chromatograms (TIC)) and full MS scan spectra (*m*/*z* 100–1500) at a retention time of 14.88 min obtained for (**a**) an Arabica coffee sample from Brazil, (**b**) a Robusta–Arabica coffee from India and (**c**) a Robusta coffee sample from Uganda.

A total of 306 coffee samples, distributed into three study cases, were analyzed after brewing the coffee using the LC-HRMS method. As a non-targeted approach was intended, a universal chromatographic separation was applied to obtain the richest instrumental responses. Hence, LC-HRMS fingerprints were registered in full MS scan mode (*m*/*z* range 100–1500). For illustration, Figure 1 shows the LC-HRMS metabolomic fingerprints (total ion chromatograms (TIC)) and representative HRMS full scan spectra (at 14.88 min) for three coffee samples (Arabica coffee from Brazil, Robusta–Arabica coffee from India and Robusta coffee from Uganda) belonging to set 1 (Section 3.3). 

TICs in Figure 1 show the total signal response from all the ions eluting at each retention time. From them, the LC-HRMS metabolomic fingerprints are an even more complex system, giving place to more than 1000 features per sample (between 1381 and 1941, depending on the samples). Although these TIC plots seem similar, subtle differences related to the number of peaks and their relative intensities can be observed. For example, at ca. 15 min, the three coffees have a very intense peak, possibly in which several compounds coelute. In addition, several characteristic signals in the range from 2 to 5 min are also observed. Another noticeable peak signal elutes at 11.3 min, also with higher intensities in Arabica and Robusta-Arabica samples. In contrast, the peaks in the range from 17 to 19 min depict higher intensities in Robusta coffees. Other less intense common signals are detected around the retention time of 9 min or in the range from 20 to 32 min. More interestingly, some signals seem to be specific for some coffee types, such as those observed at retention times of 24 and 28 min for Robusta and Arabica coffees, respectively. In any case, it should be mentioned that the obtained LC-HRMS fingerprints seem to be reproducible within the samples belonging to the same category; thus, they are useful as chemical descriptors to address sample classification by chemometrics. 

### 2.2. Sample Exploration and Classification According to the Coffee Variety

**Figure 2:**
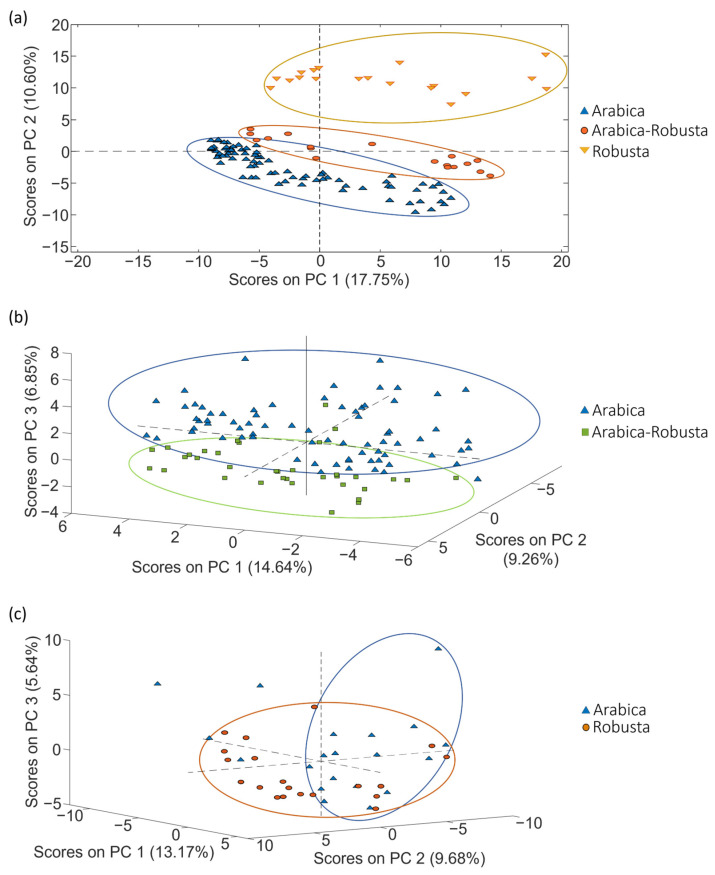
PCA score plots obtained when corrected non-targeted LC-HRMS metabolomic fingerprints were used as sample chemical descriptors to study coffee samples of (**a**) set 1 (score plot of PC1 vs. PC2), (**b**) set 2 (score plot of PC1 vs. PC2 vs. PC3) and (**c**) set 3 (score plot of PC1 vs. PC2 vs. PC3), according to the coffee variety.

The potential of LC-HRMS fingerprints as discriminative chemical markers for the classification of coffee was assessed using PCA. The first goal of PCA was to examine the behavior of quality control samples (QCs). QCs were blended mixtures containing a portion of each coffee extract within each set of samples, as explained in Section 3.3. The corresponding data matrix (X-matrix) for each study case included their respective QCs. These matrices consisted of intensity signals detected at a specific *m*/*z* value and retention time (i.e., the so-called features), and their dimensions were *n* × *f*, with *n* being the number of samples (including the QCs) and *f* being the number of features. An autoscaling preprocessing was applied to ensure that all variables were equally weighted. In the obtained PCA score plots, QCs did not appear grouped but displayed a linear distribution trend depending on their injection order within the sequence. This behavior indicates that something affected the LC-HRMS fingerprinting signal throughout the sample sequence. In fact, the QCs’ signal decreased from the beginning of the sample sequence to the end, probably because the electrospray source became dirty over time, and the sensitivity decreased correspondingly. As a consequence, the fingerprinting data matrix needs to be corrected considering the variation in signal intensity observed for the QCs to ensure a rigorous interpretation of chemometric results when undertaking classification and authentication studies. Given that the QC samples are injected regularly throughout the sample sequence, the sensitivity decay of each sample fingerprint was compensated using the nearest QCs injected in the sequence (each feature data were divided by the corresponding value from the nearest QCs; also, each QC data were divided by itself, resulting in fingerprinting variables normalized to 1). As an example, Figure 2 shows the PCA score plot of PC1 vs. PC2 when using the corrected LC-HRMS metabolomic fingerprints for set 1, set 2 and set 3 of coffee samples (see Section 3.3), displayed by labeling the samples according to the coffee variety. As expected, the correction provided more compact clusters for sample types. 

Considering the PCA distribution of coffee, in general, samples with similar attributes (variety) tended to be grouped. As shown in Figure 2, a very acceptable discrimination among the samples was achieved.

**Figure 3:**
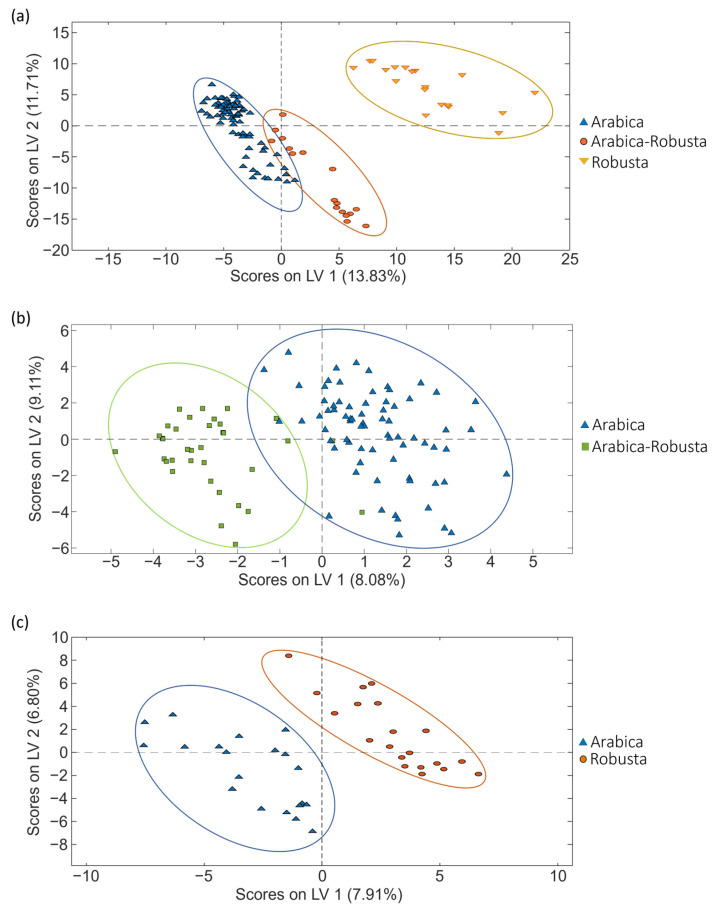
PLS-DA score plots (LV1 vs. LV2) obtained when corrected non-targeted LC-HRMS metabolomic fingerprints were used as sample chemical descriptors to study coffee samples of (**a**) set 1, (**b**) set 2 and (**c**) set 3, according to the coffee variety.

As previously mentioned, PCA is a non-supervised exploratory method useful to study the initial behavior of the analyzed samples. However, as depicted in Figure 3, to improve sample group discrimination, the corrected data matrices for each coffee set were also used to address coffee classification according to the three sample sets according to the coffee variety by PLS-DA. 

When focusing on the classification regarding coffee varieties, excellent sample classifications were accomplished for all the sets studied, as depicted in Figure 3. Perfect differentiation was always obtained between pure Arabica and pure Robusta samples (independently of the geographical origin of the samples), and very acceptable sample discrimination was also observed for blended varieties (mixtures of Arabica–Robusta). These results are also confirmed by the figures of merit shown in Table 1, showing good sensitivity and specificity values (>94.3% and >94.3%, respectively), as well as classification errors below 3.5%.

**Figure 4:**
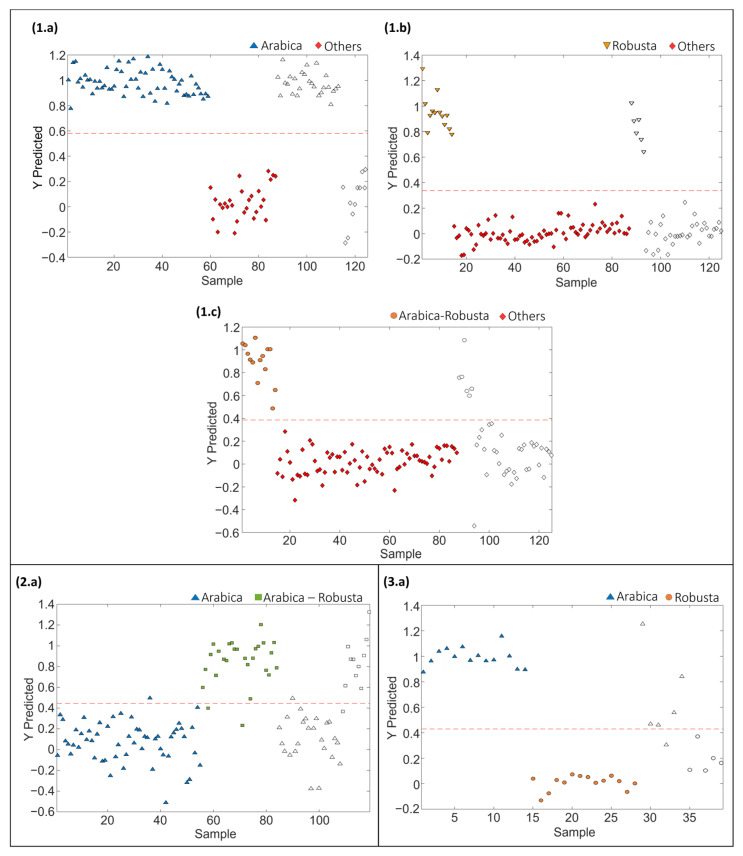
Paired PLS-DA classification plots of Y predicted vs. samples for set 1: (**1.a**) Arabica vs. Others, (**1.b**) Robusta vs. Others, (**1.c**) Arabica–Robusta mixture vs. Others, for set 2: (**2.a**) Arabica vs. Arabica–Robusta mixture, and for set 3: (**3.a**) Arabica vs. Robusta. Filled and empty symbols correspond to calibration and prediction sets, respectively. Red lines represent the threshold between classes.

PLS-DA paired models were also evaluated and validated to address the classification rates when considering a single sample class against all the others. Each paired PLS-DA model was built using 70% of the samples, randomly selected, as the calibration set and the remaining 30% as the test set of “unknown” samples for validation purposes. The results obtained for the different coffee varieties involved in sets 1, 2 and 3 are shown in Figure 4. Moreover, Table 2 provides the optimal number of LVs for each paired classification model, as well as the values of accuracy, sensitivity, specificity, and classification error achieved for both calibration and prediction steps for sets 1, 2 and 3, respectively. 

The proposed LC-HRMS fingerprinting methodology seems to be suitable for preventing blended coffee frauds, as demonstrated by the good classification performance attained, according to the variety of coffee, with paired PLS-DA model accuracy, sensitivity and specificity higher than 96.5%, 92.6% and 92.6%, respectively, and classification errors lower than 4.6%. In the case of model prediction, accuracy, sensitivity, specificity and classification errors >90.9%, >83.3%, >90.9% and <8.3%, respectively, were obtained. 

### 2.3. Sample Exploration and Classification According to the Coffee Geographical Production Region

To carry out the exploration and classification of samples based on their geographical production region, the same approach described in the preceding section was followed. 

**Figure 5:**
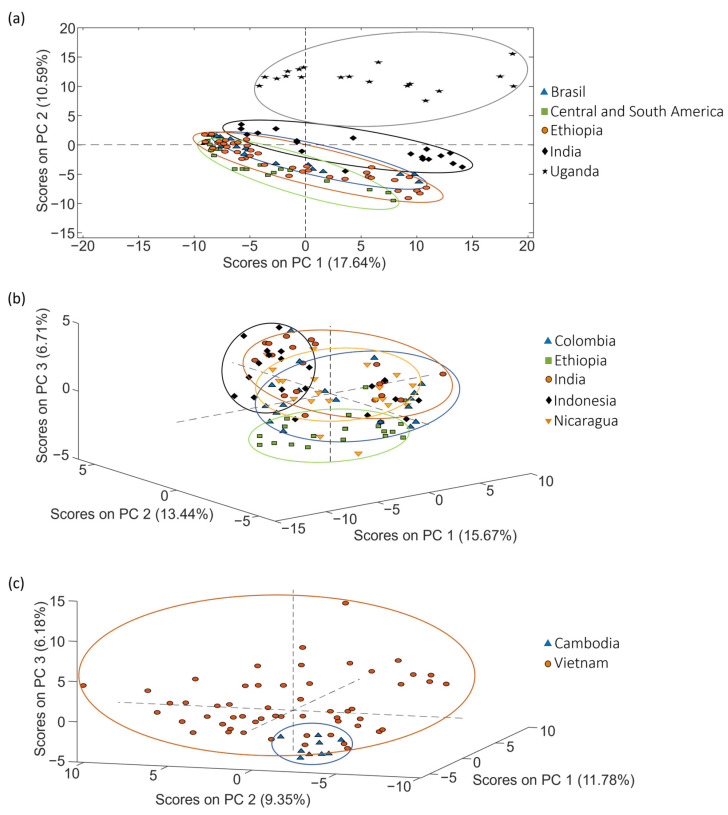
PCA score plots obtained when corrected non-targeted LC-HRMS metabolomic fingerprints were used as sample chemical descriptors to study coffee samples of (**a**) set 1 (score plot of PC1 vs. PC2), (**b**) set 2 (score plot of PC1 vs. PC2 vs. PC3) and (**c**) set 3 (score plot of PC1 vs. PC2 vs. PC3), according to the coffee geographical production region.

The exploration of samples, as illustrated in Figure 5 through PCA score plots, provides a visual representation of how the samples are distributed based on their geographical production region but, interestingly, this distribution is significantly influenced by the coffee varieties. This influence contributes to the overlap observed among different groups of samples, particularly noticeable in sets 1 and 2. 

**Figure 6:**
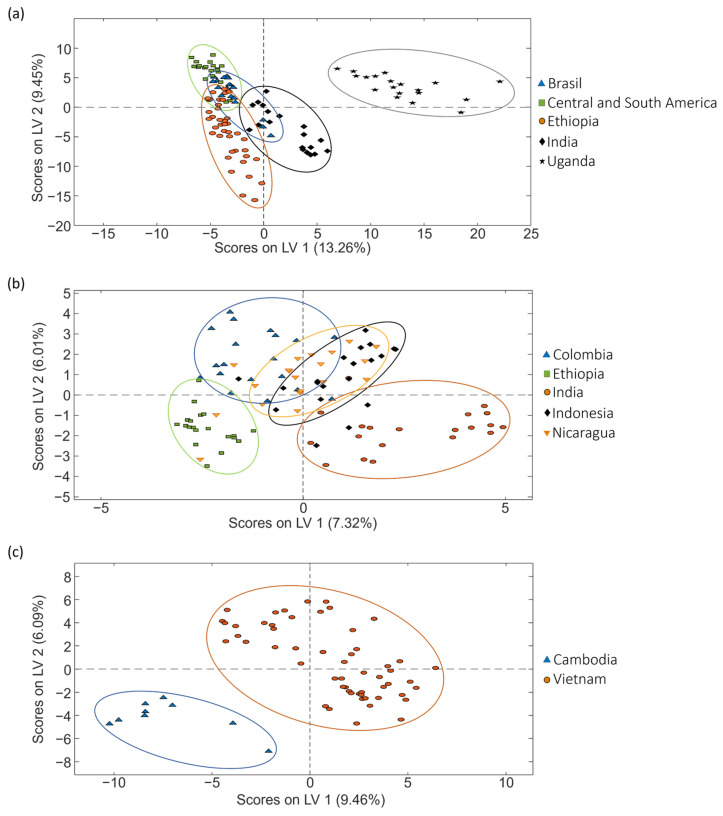
PLS-DA score plots (LV1 vs. LV2) obtained when corrected non-targeted LC-HRMS metabolomic fingerprints were used as sample chemical descriptors to study coffee samples of (**a**) set 1, (**b**) set 2 and (**c**) set 3, according to the geographical production region.

Subsequently, PLS-DA was employed to classify the samples and attempt to enhance the distribution as observed in the previous PCA score plots (Figure 5). The better obtained PLS-DA classification score plots obtained for sets 1, 2 and 3 are shown in Figure 6. 

Focusing on the classification of coffee samples based on their geographical origin, the proposed approach provides acceptable results. In all the cases, the samples are grouped according to their geographical production region, with more or less discrimination among the different sample groups depending on the complexity of the sample set. For example, in set 1 (Figure 6a) including five sample classes, it can be observed the proposed fingerprints discriminate the samples from different countries. In fact, when considering the proposed multiclass PLS-DA model (Table 3), acceptable results were obtained. 

The results worsened for set 2 (Figure 6b). In any case, again samples clustered according to their corresponding country of production, albeit with some observed overlapping in certain instances. When considering the proposed multiclass PLS-DA model (Table 3), sensitivities and specificities were higher than 80% and 51.9%, respectively, and classification errors were lower than 34.1%. 

The results clearly improved for set 3 (Figure 6c), with clear discrimination between the Cambodian and Vietnamese samples. Despite the lower complexity of this case (with only two sample groups involved), coffee growing conditions are quite similar between these two groups due to their geographical proximity (and climatic conditions) in comparison to the geographical production regions addressed in the other sets of samples. Cross-validated multiclass predictions (Table 3) provide 100% sensitivity and specificity values, with 100% sample classification rates.

As has been performed for the case of sample classification based on coffee varieties, to evaluate and validate PLS-DA classifications, PLS-DA paired models were executed. The results obtained for the different coffee origins involved in sets 1, 2 and 3, are shown in Figure S1 (Supplementary Materials). Moreover, Table S1 (Supplementary Materials) provides the optimal number of LVs, accuracy, sensitivity, specificity, and classification error for each paired classification model and for both calibration and prediction steps for sets 1, 2 and 3, respectively. 

As shown in Table S1, the obtained results for calibration are favorable, with accuracy, sensitivity and specificity values higher than 89.2%, 84.6% and 89.9%, respectively, and calibration errors below 12.7%. However, for PLS-DA prediction, sets 1 and 2 presented values capable of improvement, especially in cases such as Brazil (set 1), or Indonesia and Nicaragua (set 2). In contrast, for set 3, the accuracy, sensitivity and specificity values were 100%, as expected. 

In response to the results obtained for sets 1 and 2, a PLS-DA classification tree was designed, in which the most different class was modeled versus a diverse group that integrates all the others to recognize (and separate) all the belonging samples in both calibration and prediction sets. The multiclass pool was analyzed with another paired model to separate the next most different class from the other. The process was repeated until all classes were separated from each other. This methodology involved a stepwise exclusion of sample groups based on their distinctive distribution patterns within each set. For example, in the case of set 1, the initial model excluded Ugandan samples as they exhibited perfect discrimination from the remaining samples (probably for being 100% Robusta samples). Subsequently, the PLS-DA model was validated in pairs: Uganda vs. Others. Then, with “Others” another classification was performed, where Ethiopia samples were the most differentiated ones. So, this process was repeated for the remaining samples with their PLS-DA validation models corresponsive, until achieving a comprehensive classification of all sample types within each set. The scheme of the sequential elimination of groups in the PLS-DA tree model for the classification of samples from sets 1 and 2 according to their geographical production region is shown in Figure S2 (Supplementary Materials).

**Figure 7:**
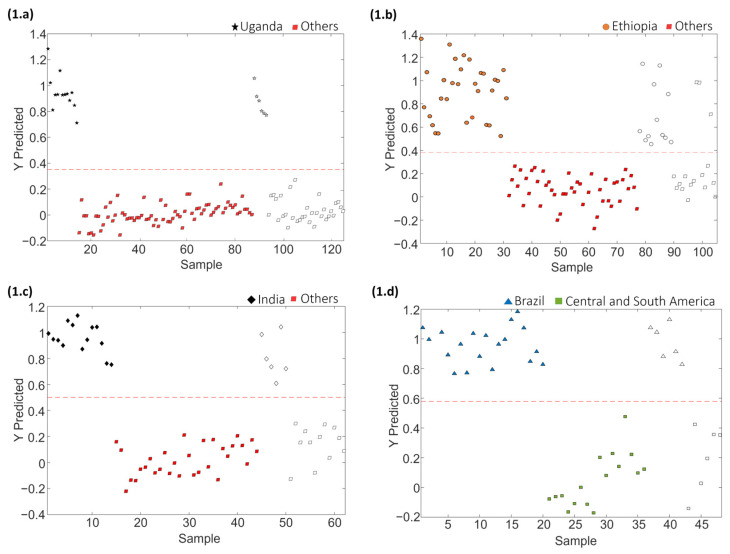
Paired PLS-DA plots of Y predicted vs. samples for set 1 within the tree classification framework: (**1.a**) Uganda vs. Others, (**1.b**) Ethiopia vs. Others, (**1.c**) India vs. Others, and (**1.d**) Brazil vs. Central and South America. Filled and empty symbols correspond to calibration and prediction sets, respectively. Red lines represent the threshold between classes.

Figure 7 depicts the sequential validation models obtained based on the order of sample group classification for set 1. Similar information is provided for coffee set 2 in Figure S3 (Supplementary Materials). Furthermore, Table 4 provides the obtained values of the optimal number of LVs, accuracy, sensitivity, specificity and errors for calibration and prediction for each PLS-DA paired validation model within the classification tree. 

As shown in Table 4, the approach of constructing PLS-DA models in a tree structure has proven effective, manifesting notably enhanced classification values across the majority of cases, obtaining accuracy, sensitivity and specificity values higher than 92.5%, 92.9% and 92.3%, respectively, and classification errors lower than 7.4% for calibration, and higher than 91.7%, 83.3% and 81.3%, and lower than 9.3%, respectively, for prediction.

### 2.4. Sample Exploration and Classification According to the Coffee Roasting Degree

**Figure 8:**
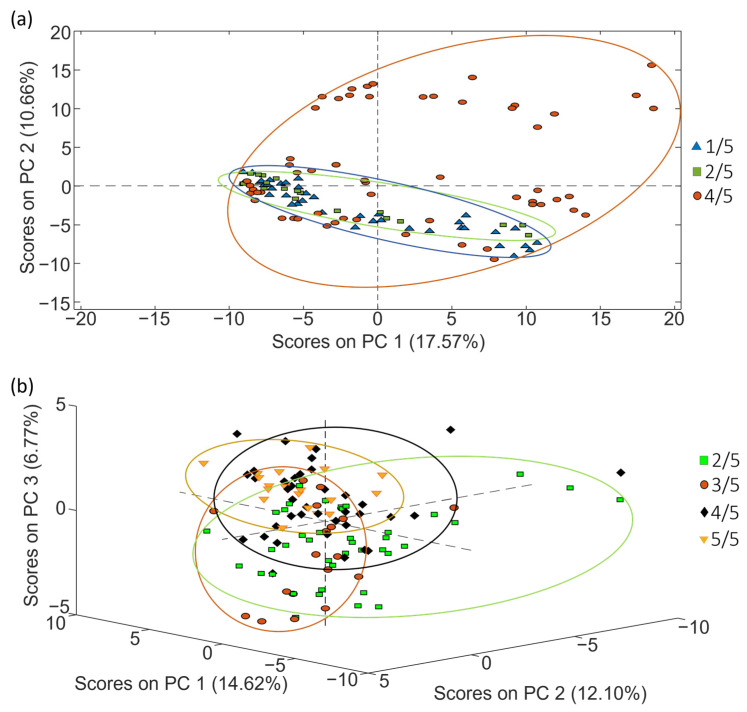
PCA score plots obtained when corrected non-targeted LC-HRMS metabolomic fingerprints were used as sample chemical descriptors to study coffee samples of (**a**) set 1 (score plot of PC1 vs. PC2) and (**b**) set 2 (score plot of PC1 vs. PC2 vs. PC3), according to the coffee roasting degree.

The exploration and classification of samples based on roasting degree followed the procedure outlined in the preceding section. The PCA score plots in Figure 8 show the distribution of samples considering their roasting degree, highlighting, once more, a notable influence of coffee varieties that contributes to the sample group overlapping. 

**Figure 9:**
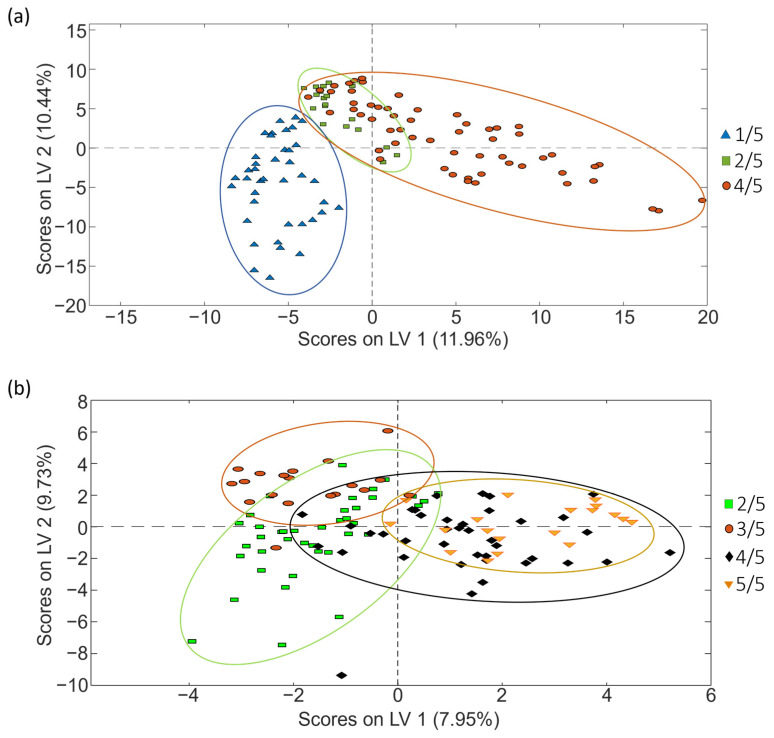
PLS-DA score plots (LV1 vs. LV2) obtained when corrected non-targeted LC-HRMS metabolomic fingerprints were used as sample chemical descriptors to study coffee samples of (**a**) set 1 and (**b**) set 2, according to the roasting degree.

Subsequently, PLS-DA was executed to carry out the classification of the samples with the aim of improving the distribution observed in the previous PCA scores plots (Figure 8). The better obtained PLS-DA classifications obtained for sets 1 and 2 are shown in Figure 9. 

As Figure 9 shows, the proposed fingerprints resulted in also being acceptable sample chemical descriptors to accomplish coffee characterization and classification based on the roasting degree for sets 1 and 2. However, depending on the case, the obtained sample groups are more or less discriminated. In any case, acceptable values for sensitivity, specificity and classification errors were also obtained (Table 5). 

As was performed for the case of sample classification based on coffee varieties and geographical origin, to evaluate and validate PLS-DA classifications, PLS-DA paired models were executed. The results obtained for the different coffee involved in sets 1, 2 and 3, are shown in Figure S4 (Supplementary Materials). In addition, Table S2 (Supplementary Materials) provides the optimal number of LVs for each paired classification model, as well as the values of accuracy, sensitivity, specificity and classification errors achieved for both calibration and prediction steps for sets 1 and 2, respectively.

As shown in Table S2, the obtained results for calibration are favorable, with accuracy, sensitivity and specificity values higher than 90%, 89.3% and 90.9%, respectively, and calibration errors below 9.9%. However, for PLS-DA prediction, sets 1 and 2 presented values capable of improvement, especially in cases such as the 4/5 roasting degree in set 1.

The PLS-DA classification tree was again assessed for the classification of samples from sets 1 and 2 according to their roasting degree. The scheme of the sequential elimination of groups in the tree model is shown in Figure S5 (Supplementary Materials).

**Figure 10:**
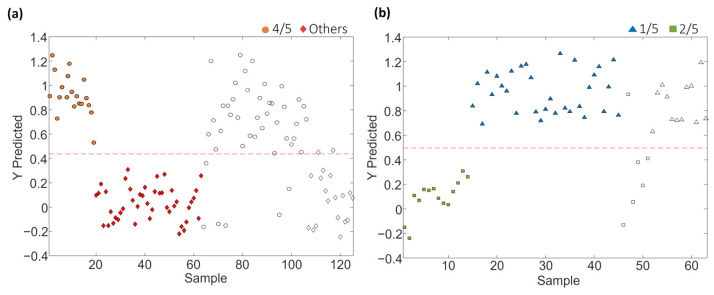
Paired PLS-DA plots of Y predicted vs. samples for set 1 within the tree classification framework: (**a**) 4/5 vs. Others and (**b**) 1/5 vs. 2/5. Filled and empty symbols correspond to calibration and prediction sets, respectively. Red lines represent the threshold between classes.

Figure 10 depicts the sequential validation models obtained for set 1. Similar information is provided for coffee set 2 in Figure S6 (Supplementary Materials). Furthermore, Table 6 provides the obtained values of accuracy, sensitivity, specificity and errors for calibration and prediction, as well as the optimal number of LVs for each PLS-DA paired validation model within the tree classification framework. 

As shown in Table 6, the approach of constructing PLS-DA models in a tree structure has proven effective, enhancing classification figures across the majority of cases, obtaining accuracy, sensitivity and specificity values higher than 95.7%, 92.9% and 96.4%, respectively, and classification errors lower than 5.4% for calibration, and higher than 87%, 83.3% and 83.3%, and lower than 16.6%, respectively, for prediction.

Overall, the obtained results prove that the proposed non-targeted LC-HRMS method based on metabolomic fingerprints allows quite acceptable sample chemical descriptors to be obtained to address coffee classification based on different coffee attributes, such as geographical origin, variety and roasting degree. In addition, in most cases, similar or slightly better sample discrimination than the one previously described by non-targeted HPLC-UV [30] and HPLC-FLD [8] was observed, with the advantage that LC-HRMS fingerprints can be very useful in the future to identify possible biomarkers from loading and VIP plots if required. Anyway, this is not mandatory in authentication issues, as demonstrated in the present contribution.

### 2.5. Detection and Quantitation of Blended Coffee Adulterations by PLS

Based on the previously described results, the capability of the corrected non-targeted LC-HRMS metabolomics fingerprinting methodology to provide sample chemical descriptors for the detection and quantitation of adulterant percentages in blended coffee samples was evaluated by PLS regression. 

For that purpose, three blended coffee adulteration cases involving nearby geographical production regions and coffee variety attributes, i.e., (i) Vietnamese Robusta coffee adulterated with Cambodian coffee, (ii) Vietnamese Arabica coffee adulterated with Cambodian coffee and (iii) Vietnamese Arabica coffee adulterated with Vietnamese Robusta coffee, were studied. These three cases were selected because of the proximity between the coffee-growing geographical regions and climatic conditions, thus it was expected they would be among the most difficult coffee fraudulent practices to be detected. 

Two independent sample sets (calibration set and validation set) were employed for each adulteration case. Table 7 shows the blended percentages to build the calibration and validation sets, where X represents the original coffee and Y represents the adulterant. Each adulteration level was prepared in quintuplicate, obtaining 55 sample extracts in total for each adulteration case under study. Additionally, an extra adulterated sample at a 50% level was used as a QC solution. 

**Figure 11:**
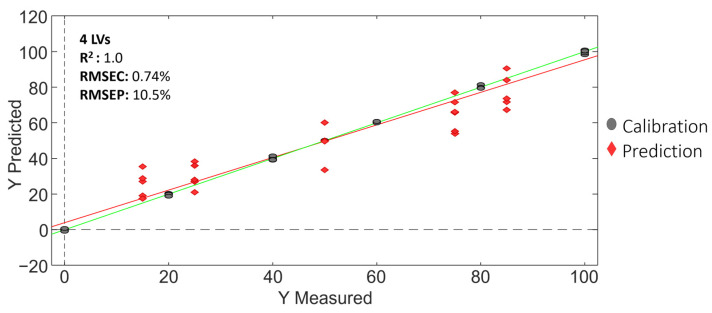
PLS regression model for the Vietnamese Robusta coffee adulterated with Cambodian coffee case.

Figure 11 shows, for illustration, the Vietnamese Robusta coffee adulterated with Cambodian coffee case, and the PLS regression model obtained. In addition, Table 8 summarizes the PLS regression results obtained for the three adulteration cases evaluated. 

As shown in Table 8, the PLS calibration for all the studied cases was very satisfactory, with good linearities (correlations higher than 0.995), and calibration and prediction errors below 0.74% and 11.6%, respectively. 

Overall, the obtained results demonstrated that the proposed non-targeted LC-HRMS metabolomics methodology is suitable to detect and quantify adulteration percentage levels in blended adulterated coffees grown in nearby geographical production regions. 

## 3. Materials and Methods

### 3.1. Chemicals and Solutions

The reagents for the preparation of the chromatographic mobile phase were HPLC grade methanol from PanReac AppliChem (Barcelona, Spain), formic acid (≥98%) from Sigma-Aldrich (St. Louis, MO, USA) and Milli-Q water from an Elix 3 coupled to a Milli-Q system (Millipore Corporation, Burlington, MA, USA) (water was filtered with a 0.22 µm nylon membrane before use).

Mineral water obtained from Eroski (Elorrio, Spain) was employed for coffee brewing.

### 3.2. LC-HRMS Instrumentation

Samples were analyzed with a UHPLC system (Dionex UHPLC instrument, Thermo Fisher Scientific, Pleasanton, CA, USA) equipped with a binary pump and an autosampler. The LC instrument was coupled to an LTQ Orbitrap Velos HRMS instrument (Thermo Fisher Scientific) with an electrospray ionization source (ESI) in negative ion mode. A reversed-phase chromatographic separation with a Kinetex^®^ C18 (100 mm × 4.6 mm, 2.6 µm partially porous particle size) column by Phenomenex (Torrance, CA, USA) was proposed, under gradient elution conditions using water with 0.1% formic acid (solvent A) and methanol (solvent B) as mobile phase components. The mobile phase flow rate was 0.4 mL/min, and the column was kept at room temperature. The gradient elution program is summarized in Table 9. The injection volume was 5 µL (full-loop mode).

ESI ionization source operated using nitrogen (purity higher than 99.98%) for the sheath, sweep and auxiliary gases at flow rates of 60, 0 and 10 a.u. (arbitrary units), respectively. The capillary temperature and ESI ionization source temperature were 350 °C and 25 °C, respectively, and an S-Lens RF level was 50 V. Orbitrap HRMS instrument was tuned and calibrated before the analysis with a commercial calibration solution (Thermo Fisher Scientific). Full MS scan mode (*m*/*z* 100–1500) with a mass resolution of 60,000 full width at half-maximum (FWHM, at *m*/*z* 200), an FTMS Full automatic gain control (AGC) Target activate of 1 × 10^6^ and a maximum injection time (IT) of 200 ms, were proposed for HRMS sample acquisition.

### 3.3. Samples and Sample Treatment

A total of 306 commercial coffee samples, grouped into three different sets (Table 10), were analyzed. 

The first two sets encompassed 120 Nespresso^®^ coffee samples purchased from supermarkets in Barcelona (Spain). These samples differed in the country of origin (geographical production region), in the coffee variety (Arabica, Robusta, or mixture of blends), and in the roasting degree (increasing from 1 to 5). The third set consisted of 66 coffee samples purchased from Vietnamese and Cambodian local supermarkets, classified into five groups depending on the coffee variety and the region of origin (no information regarding the roasting degree was available). Moreover, this set was designed to address the applicability of the proposed methodology for the classification and authentication of coffees from nearby countries produced under similar climatic conditions.

Coffees were brewed with mineral water to prevent any variation caused by the water composition. For the two first cases, the brewing process was with an espresso machine (Nespresso^®^), and always using the same final volume. On the other hand, for the third set, coffees were brewed with an Italian coffee maker (Moka pot), grinding the coffee beans when necessary. In this second procedure, ground coffee (~40 g) introduced well compressed into the Italian coffee maker was brewed with 400 mL of the mineral water using a Bunsen burner to carry out the coffee lixiviation. Coffee extracts were filtered with 0.45 µm nylon filters (Phenomenex, Alcobendas, Spain) into 2 mL glass vials which were stored at −4 °C until LC-HRMS analysis. Additionally, a QC solution was prepared for each set of samples by mixing 50 µL of each sample extract. 

Some coffee samples belonging to set 3 were also used in the adulteration studies. For this purpose, adulterated samples were blended with different amounts of other substances as previously described in Section 2.5.

### 3.4. Data Analysis

All the samples belonging to each set were randomly analyzed with the non-targeted LC-HRMS method. LC-HRMS raw chromatographic data were then processed with the MSConvert v3.0 free software. It should be considered that this work aimed to develop a method able to provide a fingerprint that reflects all the influence of any sample specific feature and thus, data were not standardized for level of roasting degree, although it obviously will influence the amount of produced chemicals. This decision was grounded in the idea of making the method simpler and, simultaneously, more applicable to all types of coffee samples. By allowing all sample characteristics to contribute to the obtained sample fingerprints, all the intrinsic complexity of the samples will be considered and it will enhance the method capability to adapt to the natural variability found in different coffee beans. 

For data simplification, a threshold peak filter of 10^4^ (absolute intensity) was applied. The resulting filtered data were processed with MZmine-2.53 free software to obtain Excel files with the chemical features detected (ion signals as a function of *m*/*z* values and retention times). In MZmine-2.53 processing, data were submitted to exact mass detection to create a mass list of individual ions for each MS spectrum throughout the chromatogram, considering a noise level of 2 × 10^4^. Then, all mass lists were filtered and the residual signals were removed using the FTMS shoulder peaks filter method. After this, a peak time range of 0.05–2 min, an *m*/*z* tolerance of 5 ppm, and an intensity threshold of 2.5 × 10^4^ were established to apply the chromatogram builder method to join the exact mass signals found in contiguous scans in a sample. Then, chromatogram deconvolution was applied to separate each detected chromatogram into individual peaks. After this, the join aligner was applied to match the exact masses detected on samples (mass tolerance of 5 ppm) with a peak retention time (tolerance of 2.5 min). Finally, the data were exported in Excel format, building a data matrix (samples × variables) where variables consisted of ion signal intensity values as a function of *m*/*z* and chromatographic retention time. Then, the resulting LC-HRMS fingerprints were filtered to eliminate those spurious features that appear occasionally in some samples but are not at all in a general pattern (they must be found in at least five samples to not be excluded from the data matrix). The resulting matrices, with a number of features of ca. 58–317 were used for PCA, PLS-DA and PLS. The SOLO 8.6 software from Eigenvector Research (Manson, WA, USA) was used for the chemometric treatment [31]. Details of the theoretical background of these statistical methodologies are addressed elsewhere [32]. 

X-data matrices to be treated by PCA and PLS-DA consisted of the obtained non-targeted LC-HRMS metabolomic fingerprints for the corresponding analyzed samples and QCs within each set of coffee samples. In all cases, normalization pretreatment concerning the overall analyte concentration was applied to provide similar weights to all samples (normalization performed according to the QCs). The Y-data matrix in the PLS-DA models defined the membership of each coffee sample in the corresponding class. Then, scatter plots of scores from principal components (PCs) were used to study the robustness of the employed method and the classification trends exhibited by the samples. In the case of PLS-DA, scatter plots of scores from latent variables (LVs) were used to study the distribution of samples. The optimal number of LVs, in both PLS-DA and PLS, was the first significant minimum point of the cross-validation (CV) errors from a Venetian blind strategy. In addition, paired PLS-DA models were assessed and validated on independent prediction sets. For that purpose, PLS-DA models were built using 70% of the sample group (randomly selected) as the calibration set, while the remaining 30% of the sample group constituted the prediction set. In addition to this, PLS-DA models in tree structure were provided in necessary cases, along with their respective validations (paired PLS-DA models). In the case of PLS, models were validated on the prediction sets by using 15%, 25%, 50%, 75% and 85% adulteration levels as described in Section 2.5.

## 4. Conclusions

In the present work, a non-targeted LC-HRMS metabolomic fingerprinting methodology has proved to provide very acceptable sample chemical descriptors to characterize, classify and authenticate coffee samples according to different attributes such as their geographical production region (even in the cases of coffees grown from nearby countries such as Vietnam and Cambodia with very similar climatic conditions), their variety (Arabica, Robusta or mixture blends) and their roasting degree, by means of chemometric methods. In addition, the proposed methodology was also able to detect fraudulent practices and quantify the adulteration percentage by PLS regression. 

Overall, the PLS-DA models achieved satisfactory classifications. Classification error rates for the different coffee varieties analyzed in coffee sample sets 1, 2 and 3 remained below 8.3%. However, for the classifications of samples based on the geographical production region and roasting degree, the performance was not as robust for sets 1 and 2. Therefore, PLS-DA models with a tree structure were employed to improve the initially obtained values, resulting in higher sensitivity and specificity percentages higher than 83.3% and 81.3%, respectively, and classification errors lower than 16.6% in predicted samples in the paired PLS-DA models.

The application of PLS in the analysis of adulterated coffee samples delivered satisfactory results, featuring commendable linearity with correlations higher than 0.995 and low calibration errors. 

The proposed methodology is very powerful thanks to the accurate mass detection provided by HRMS and the large amount of chemical information recorded for each sample using a fingerprinting approach; therefore, it could be very useful for future experiments, if required, aimed at biomarker identification, which can then be proposed as marker compounds to be monitored by targeted methodologies. However, we have demonstrated that the proposed non-targeted LC-HRMS metabolomic fingerprints can be easily employed without the requirement of compound identification (simplifying the cost and difficulty of use of the proposed methodology) to address coffee authentication issues, as well as to detect coffee fraudulent practices using blended adulterations. 

## Figures and Tables

**Table 1:** Sensitivity, specificity and classification errors by PLS-DA when studying the coffee samples according to their variety.

Class	Sensitivity (%)	Specificity (%)	Classification Errors (%)
Set 1			
Arabica	100	100	0
Arabica–Robusta mixture	100	100	0
Robusta	100	100	0
Set 2			
Arabica	98.6	94.3	3.5
Arabica–Robusta mixture	94.3	98.6	3.5
Set 3			
Arabica	100	100	0
Robusta	100	100	0

**Table 2:** Accuracy, sensitivity, specificity and classification errors obtained for calibration and prediction on paired PLS-DA models for sets 1, 2 and 3 according to their variety.

Class	LVs		Calibration				Prediction		
		Accuracy (%)	Sensitivity (%)	Specificity (%)	Classification Error (%)	Accuracy (%)	Sensitivity (%)	Specificity (%)	Classification Errors (%)
Set 1									
Arabica	3	100	100	100	0	100	100	100	0
Arabica–Robusta mixture	2	100	100	100	0	100	100	100	0
Robusta	2	100	100	100	0	100	100	100	0
Set 2									
Arabica	2	96.5	98.2	92.6	4.6	94.3	95.9	90.9	6.6
Arabica–Robusta mixture	2	96.5	92.6	98.2	4.6	94.3	90.9	95.8	6.6
Set 3									
Arabica	3	100	100	100	0	90.9	83.3	100	8.3
Robusta	3	100	100	100	0	90.9	100	83.3	8.3

**Table 3:** Sensitivity, specificity and classification errors by PLS-DA when studying the coffee samples according their geographical production region.

Class	Sensitivity (%)	Specificity (%)	Classification Errors (%)
Set 1			
Brazil	94.7	98	3.6
Central and South America	95	99	3
Ethiopia	100	98.7	0.6
India	100	100	0
Uganda	100	100	0
Set 2			
Colombia	89.5	81.3	14.6
Ethiopia	95.0	94.9	5
India	90.0	97.5	6.3
Indonesia	70.0	79.7	25.1
Nicaragua	80.0	51.9	34.1
Set 3			
Cambodia	100	100	0
Vietnam	100	100	0

**Table 4:** Accuracy, sensitivity, specificity and classification errors obtained for calibration and prediction on paired PLS-DA models for set 1 and 2 according to the geographical production region within the tree classification framework.

Class	LVs		Calibration				Prediction		
		Accuracy (%)	Sensitivity (%)	Specificity (%)	Classification Errors (%)	Accuracy (%)	Sensitivity (%)	Specificity (%)	Classification Errors (%)
Set 1									
Ethiopia	2	100	100	100	0	100	100	81.3	9.3
Brazil	2	100	100	100	0	100	100	100	0
Central and South America	2	100	100	100	0	100	100	100	0
India	2	100	100	100	0	100	100	100	0
Uganda	2	100	100	100	0	100	100	100	0
Set 2									
Colombia	3	100	100	100	0	91.7	83.3	100	8.3
Ethiopia	3	100	100	100	0	96.7	83.3	100	8.3
India	2	100	100	100	0	95.2	100	93.8	3.1
Indonesia	2	92.5	92.9	92.3	7.4	89.5	100	84.6	7.7
Nicaragua	3	100	100	100	0	91.7	100	83.3	8.3

**Table 5:** Sensitivity, specificity and classification errors by PLS-DA when studying the coffee samples according their origin region.

Class	Sensitivity (%)	Specificity (%)	Classification Errors (%)
Set 1			
1/5	100	100	0
2/5	100	98	1
4/5	98.3	100	0.9
Set 2			
2/5	72.5	75.3	26.1
3/5	89.5	87.8	11.4
4/5	79.5	84.6	17.9
5/5	94.7	89.8	7.7

**Table 6:** Accuracy, sensitivity, specificity and classification errors obtained for calibration and prediction on paired PLS-DA models for sets 1 and 2 according to the roasting degree within the tree classification framework.

Class	LVs		Calibration				Prediction		
		Accuracy (%)	Sensitivity (%)	Specificity (%)	Classification Errors (%)	Accuracy (%)	Sensitivity(%)	Specificity (%)	Classification Errors (%)
Set 1									
1/5	2	100	100	100	0	100	100	83.3	8.3
2/5	2	100	100	100	0	94.4	83.3	100	8.3
4/5	3	100	100	100	0	87.1	86	89.5	12.2
Set 2									
2/5	3	100	100	96.4	1.8	87	91.7	83.3	12.5
3/5	2	95.7	92.9	96.4	5.4	87.1	83.3	83.3	16.6
4/5	3	98	96.4	100	1.8	87	83.3	91.7	12.5
5/5	4	96.4	92.9	97.1	5	94.6	83.3	96.7	10

**Table 7:** Concentration levels of the blended coffees employed in calibration and validation sets for each adulteration case evaluated. X is the original coffee and Y the coffee considered the adulterant.

	X% (Coffee)	Y% (Adulterant)
Calibration	100	0
	80	20
	60	40
	40	60
	20	80
	0	100
Validation	15	85
	25	75
	50	50
	75	25
	85	15

**Table 8:** Evaluation of the coffee adulteration cases by PLS using corrected non-targeted LC-HRMS metabolomics fingerprints as chemical descriptors.

Pure Coffee	Adulterant	LVs	R^2^	Calibration Errors (%)	Prediction Errors (%)
Vietnamese Arabica Coffee	Cambodian coffee	4	1.0	0.74	10.5
Vietnamese Robusta Coffee	Cambodian coffee	2	0.995	2.7	10.8
Vietnamese Arabica Coffee	Vietnamese Robusta Coffee	3	0.999	1.1	11.6

**Table 9:** LC-HRMS elution program.

Time (min)	Methanol (%)	Elution Mode
0	3	Initial conditions
30	75	Linear gradient
32	95	Linear gradient
34	95	Isocratic step
34.2	3	Linear gradient
40	3	Isocratic (column re-equilibration)

**Table 10:** Description of the analyzed coffee samples.

Commercial Name	Number of Samples	Coffee Variety	Origin Region	Roasting Degree
		Set of samples number 1		
Arabica Ethiopia Harrar	20	Arabica	Ethiopia	1/5
Bukeela	20	Arabica–Arabica Mixture	Ethiopia	1/5
Dulsao	20	Arabica	Brazil	2/5
Arpeggio	20	Arabica	Central and South-America	4/5
Indriya	20	Arabica–Robusta Mixture	India	4/5
Robusta Uganda	20	Robusta	Uganda	4/5
		Set of samples number 2		
Master Origin Colombia	20	Arabica	Colombia	3/5
Master Origin Ethiopia	20	Arabica	Ethiopia	2/5
Master Origin India	20	Arabica–Robusta Mixture	India	5/5
Master Origin Nicaragua	20	Arabica	Nicaragua	2/5
Master Origin Indonesia	20	Arabica	Indonesia	4/5
Paris Black	20	Arabica–Robusta Mixture	Unknown origin	4/5
		Set of samples number 3		
-	20	Arabica	Vietnam	Unknown
-	20	Robusta	Vietnam	Unknown
-	10	Arabica–Robusta Mixture	Vietnam	Unknown
-	6	Unknown	Vietnam	Unknown
-	10	Unknown	Cambodia	Unknown

## Data Availability

Data are available upon request to the authors.

## Supplementary Materials

The following supporting information can be downloaded at: https://www.mdpi.com/article/10.3390/molecules29010232/s1, Table S1. Accuracy, sensitivity, specificity and classification error obtained for calibration and prediction on paired PLS-DA models for set 1, 2 and 3 according to the geographical production region.; Table S2. Accuracy, sensitivity, specificity and classification error obtained for calibration and prediction on paired PLS-DA models for set 1 and 2 according to the roasting degree; Figure S1. Paired PLS-DA plots of Y predicted vs. samples for set 1: (1.a) Uganda vs. Others, (1.b) India vs. Others, (1.c) Brazil vs. Others, (1.d) Ethiopia vs. Others, (1.e) Central and South America vs. Others, for set 2: (2.a) Colombia vs. Others, (2.b) Ethiopia vs. Others, (2.c) India vs. Others, (2.d) Indonesia vs. Others, (2.e) Nicaragua vs. Others, and for set 3: (3.a) Cambodia vs. Vietnam. Filled and empty symbols correspond to calibration and prediction sets, respectively. Red lines represent the threshold between classes; Figure S2. Classification scheme based on the geographical production region of coffee in tree structure for (a) set 1 and (b) set 2; Figure S3. Paired PLS-DA plots of Y predicted vs. samples for set 2 within the tree classification framework: (1.a) Ethiopia vs. Others, (1.b) India vs. Others, (1.c) Indonesia vs. Others, and (1.d) Colombia vs. Nicaragua. Filled and empty symbols correspond to calibration and prediction sets, respectively. Red lines represent the threshold between classes; Figure S4. Paired PLS-DA plots of Y predicted vs. samples for set 1: (1.a) 1/5 vs. Others, (1.b) 2/5 vs. Others, and (1.c) 4/5 vs. Others, for set 2: (2.a) 2/5 vs. Others, (2.b) 3/5 vs. Others, (2.c) 4/5 vs. Others and, (2.d) 5/5 vs. Others. Filled and empty symbols correspond to calibration and prediction sets, respectively. Red lines represent the threshold between classes; Figure S5. Classification scheme based on the roasting degree of coffee in tree structure for (a) set 1 and (b) set 2; Figure S6. Paired PLS-DA plots of Y predicted vs. samples for set 2 within the tree classification framework: (a) 5/5 vs. Others, (b) 3/5 vs. Others and, (c) 2/5 vs. 4/5. Filled and empty symbols correspond to calibration and prediction sets, respectively. Red lines represent the threshold between classes.
