# Supplementary material for: Liquid Chromatography–High-Resolution Mass Spectrometry (LC-HRMS) Fingerprinting and Chemometrics for Coffee Classification and Authentication
Source: Molecules. 2023 Dec 31;29(1):232. doi: 10.3390/molecules29010232 (PMC10780290; doi:10.3390/molecules29010232)
Supplement: Supplementary file 1 [file molecules-29-00232-s001.zip › molecules-2782242-supplementary.pdf]

Supplementary Material

Liquid Chromatography–High-Resolution Mass Spectrometry (LC-HRMS) Fingerprinting and Chemometrics for Coffee Classification and Authentication

Nerea Núñez [1,2*], Javier Saurina [1,2] and Oscar Núñez [1,2,3]

[1] Department of Chemical Engineering and Analytical Chemistry, University of Barcelona, Martíi Franquès 1-11, E08028 Barcelona, Spain; xavi.saurina@ub.edu (J.S.); oscar.nunez@ub.edu (O.N.)
[2] Research Institute in Food Nutrition and Food Safety, University of Barcelona, Recinte Torribera, Av. Prat de la Riba 171, Edifici de Recerca (Gaudí), Santa Coloma de Gramenet, E08921 Barcelona, Spain
[3] Serra Húnter Fellow, Departament de Recerca i Universitats, Generalitat de Catalunya, Via Laietana 2, E08003 Barcelona, Spain
* Correspondence: nereant7@gmail.com

Table S1. Accuracy, sensitivity, specificity and classification error obtained for calibration and prediction on paired PLS-DA models for set 1, 2 and 3 according to the geographical production region.

Class	LVs	Calibration				Prediction			
		Accuracy (%)	Sensitivity (%)	Specificity (%)	Classification Error (%)	Accuracy (%)	Sensitivity (%)	Specificity (%)	Classification Error (%)
Set 1									
Ethiopia	2	100	100	94.7	2.6	100	100	84	8
Brazil	2	100	100	98.6	0.7	94.7	66.7	87.5	22.9
Central and South America	2	100	100	93.1	3.4	100	100	90.6	4.7
India	2	100	100	100	0	94.7	66.7	100	16.7
Uganda	2	100	100	100	0	100	100	100	0
Set 2									
Colombia	2	89.2	84.6	90	12.7	97.2	83.3	100	8.3
Ethiopia	2	98.7	92.9	98.6	4.3	94.4	100	90	5
India	2	95.2	92.9	95.7	5.7	94.6	100	93.3	3.3
Indonesia	2	94.3	92.9	92.8	7.2	86.1	50	73.3	38.3
Nicaragua	3	90.2	92.9	89.9	8.6	69.4	16.7	80	51.7
Set 3									
Cambodia	2	100	100	100	0	100	100	100	0
Vietnam	2	100	100	100	0	100	100	100	0

Table S2. Accuracy, sensitivity, specificity and classification error obtained for calibration and prediction on paired PLS-DA models for set 1 and 2 according to the roasting degree.

**Class**	**LVs**	**Calibration**				**Prediction**			
		**Accuracy (%)**	**Sensi tivity (%)**	**Speci ficity (%)**	**Classification Error (%)**	**Accuracy (%)**	**Sensi tivity (%)**	**Speci ficity (%)**	**Classification Error (%)**
Coffee geographical production region									
1/5	3	100	100	94.7	2.6	100	100	92	4
2/5	3	97.6	92.9	97.3	4.9	100	100	93.8	3.1
4/5	2	100	100	97.7	1.1	89.7	78.9	100	42.1
Coffee variety									
2/5	3	90	89.3	90.9	9.9	75	91.7	62.5	22.9
3/5	3	98.2	100	98	1	91.7	66.7	96.7	18.3
4/5	3	96.1	96.4	94.5	4.5	63.9	58.3	75	33.3
5/5	3	97.6	91.7	98.6	4.9	83.3	66.7	80	26.7

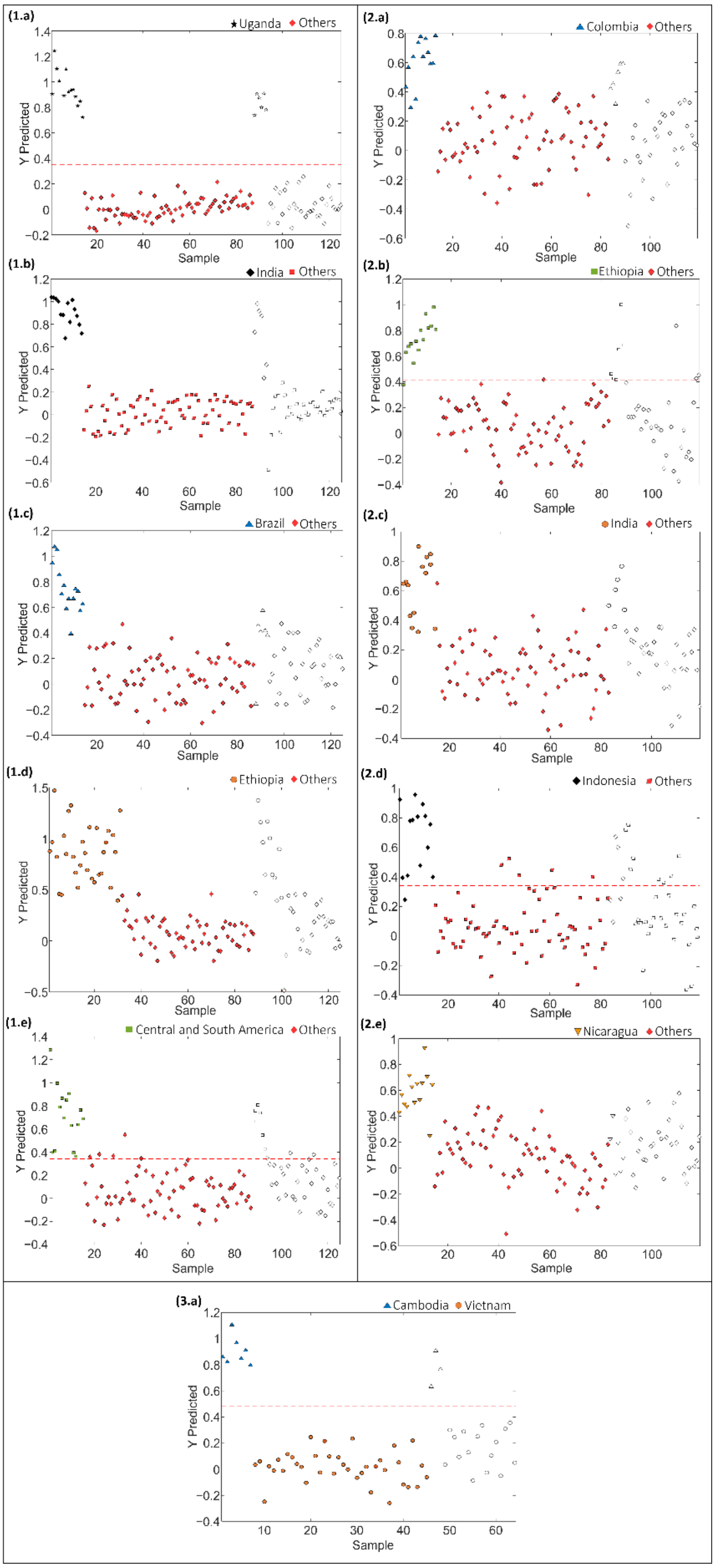

Figure S1. Paired PLS-DA plots of Y predicted *vs.* samples for set 1: (1.a) Uganda *vs.* Others, (1.b) India *vs.* Others, (1.c) Brazil *vs.* Others, (1.d) Ethiopia *vs.* Others, (1.e) Central and South America *vs.* Others, for set 2: (2.a) Colombia *vs.* Others, (2.b) Ethiopia *vs.* Others, (2.c) India *vs.* Others, (2.d) Indonesia *vs.* Others, (2.e) Nicaragua *vs.* Others, and for set 3: (3.a) Cambodia *vs.* Vietnam. Filled and empty symbols correspond to calibration and prediction sets, respectively. Red lines represent the threshold between classes.

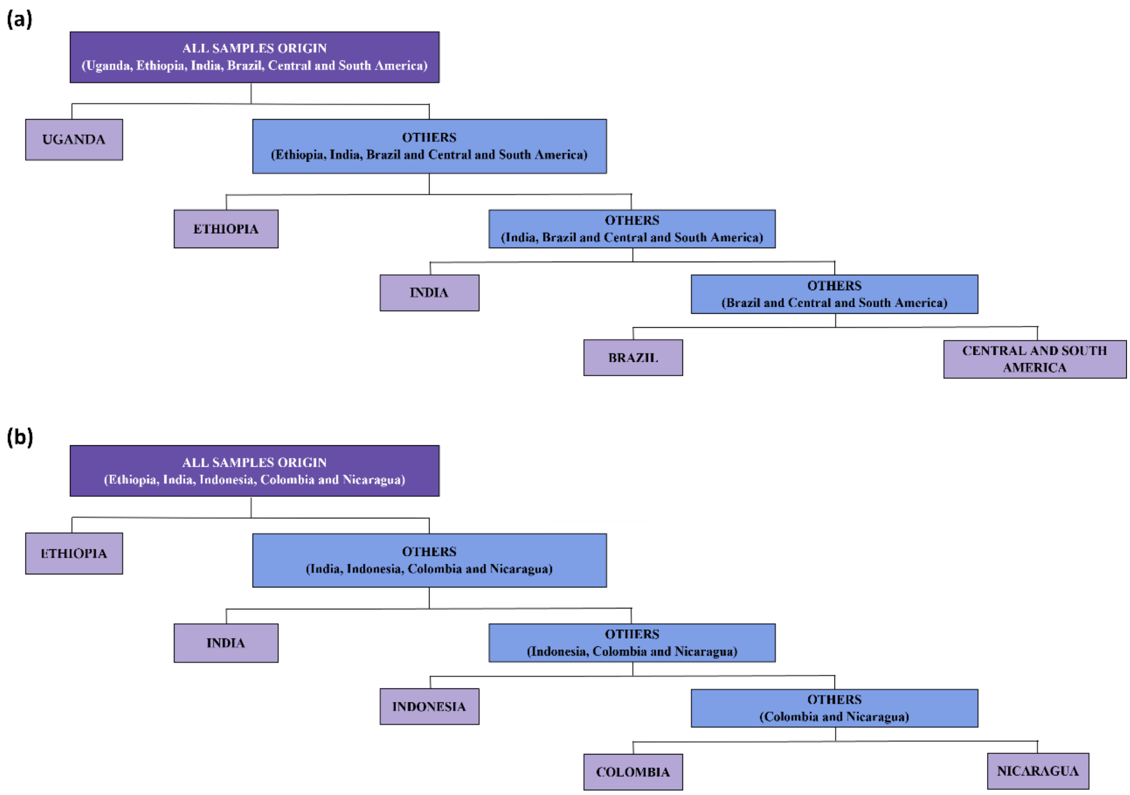

Figure S2. Classification scheme based on the geographical production region of coffee in tree structure for (a) set 1 and (b) set 2.

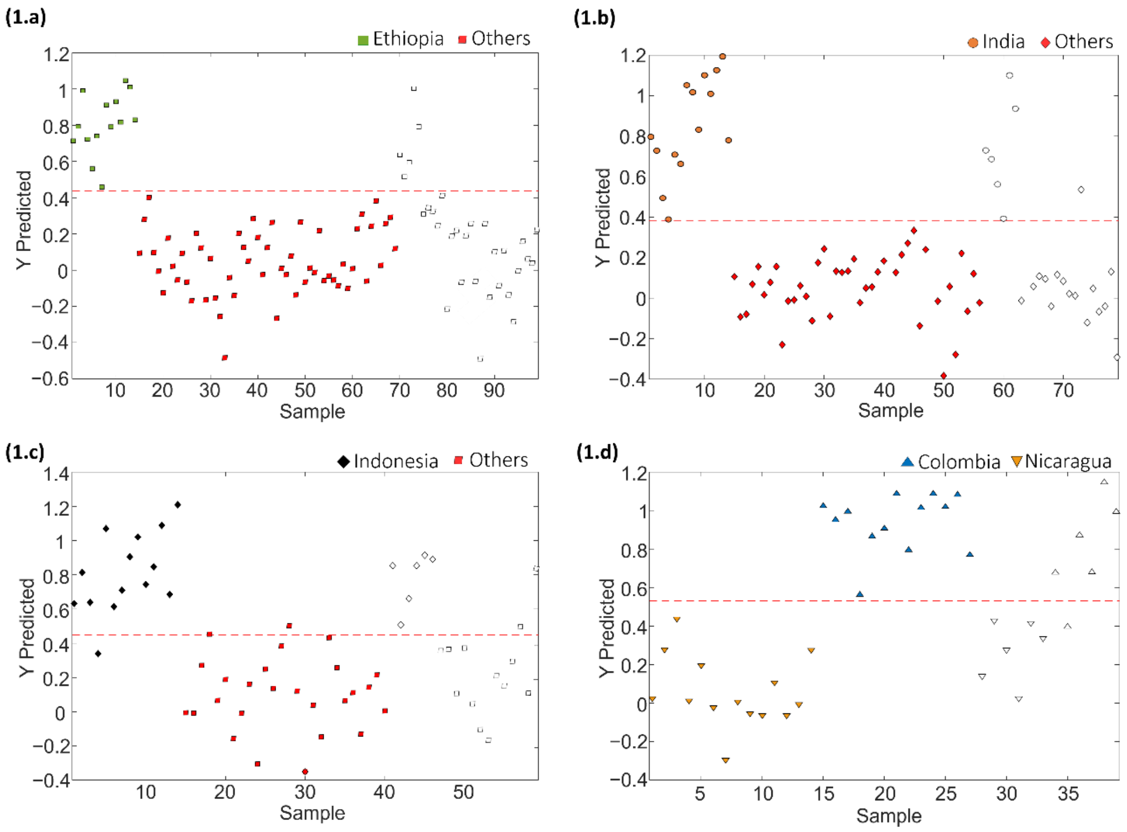

Figure S3. Paired PLS-DA plots of Y predicted *vs.* samples for set 2 within the tree classification framework: (1.a) Ethiopia *vs.* Others, (1.b) India *vs.* Others, (1.c) Indonesia *vs.* Others, and (1.d) Colombia *vs.* Nicaragua. Filled and empty symbols correspond to calibration and prediction sets, respectively. Red lines represent the threshold between classes.

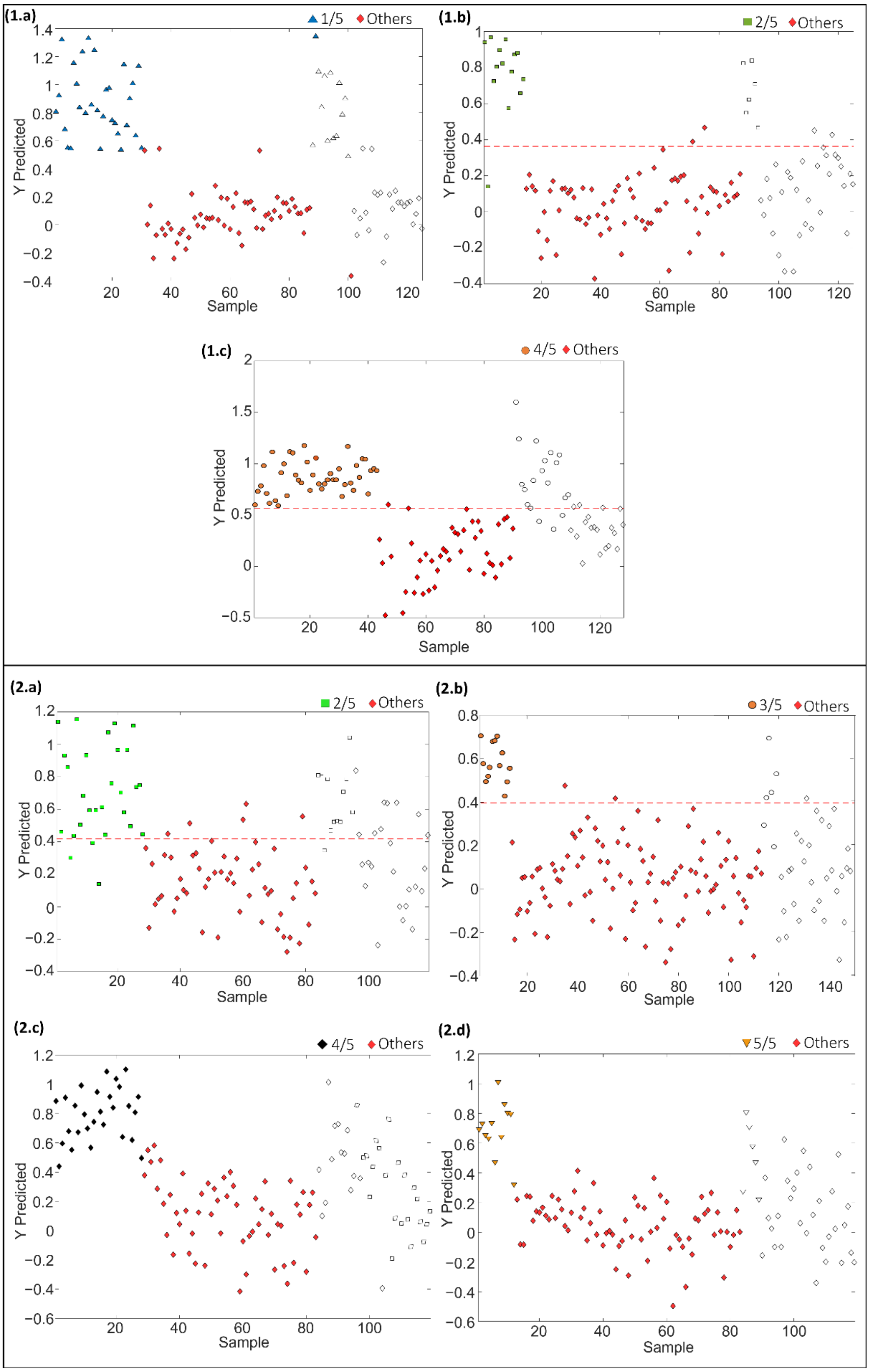

Figure S4. Paired PLS-DA plots of Y predicted *vs.* samples for set 1: (1.a) 1/5 *vs.* Others, (1.b) 2/5 *vs.* Others, and (1.c) 4/5 *vs.* Others, for set 2: (2.a) 2/5 *vs.* Others, (2.b) 3/5 *vs.* Others, (2.c) 4/5 *vs.* Others and, (2.d) 5/5 *vs.* Others. Filled and empty symbols correspond to calibration and prediction sets, respectively. Red lines represent the threshold between classes.

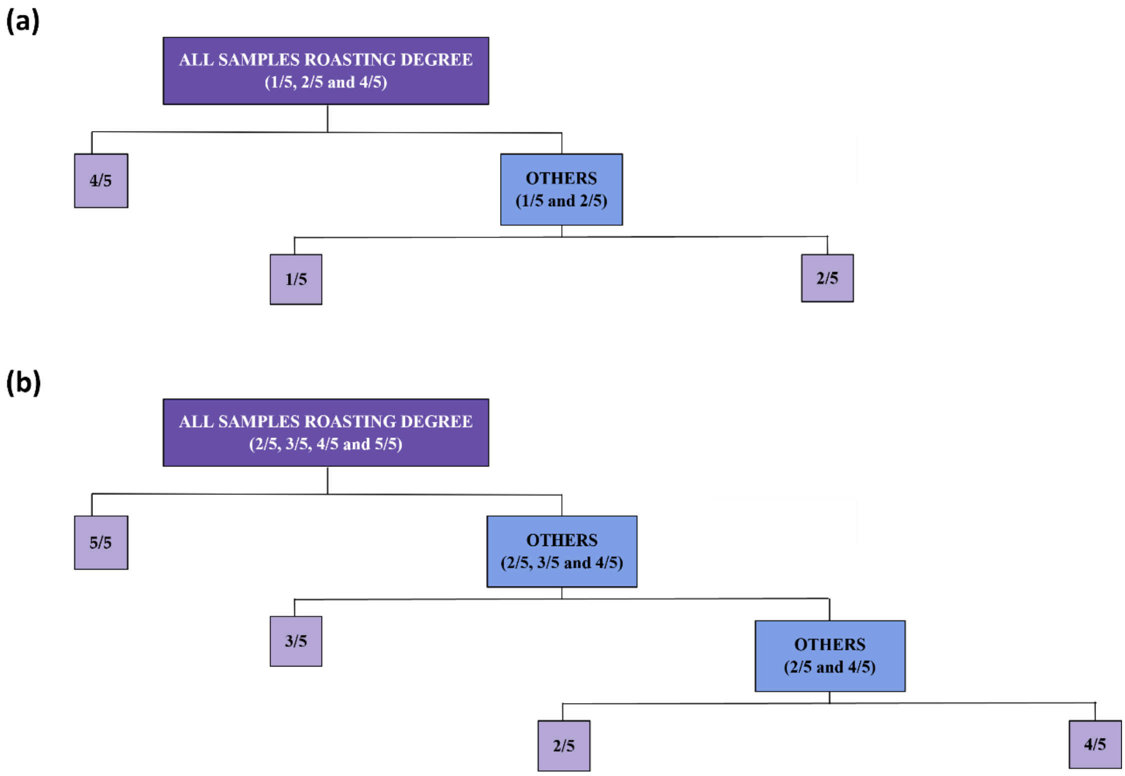

Figure S5. Classification scheme based on the roasting degree of coffee in tree structure for (a) set 1 and (b) set 2.

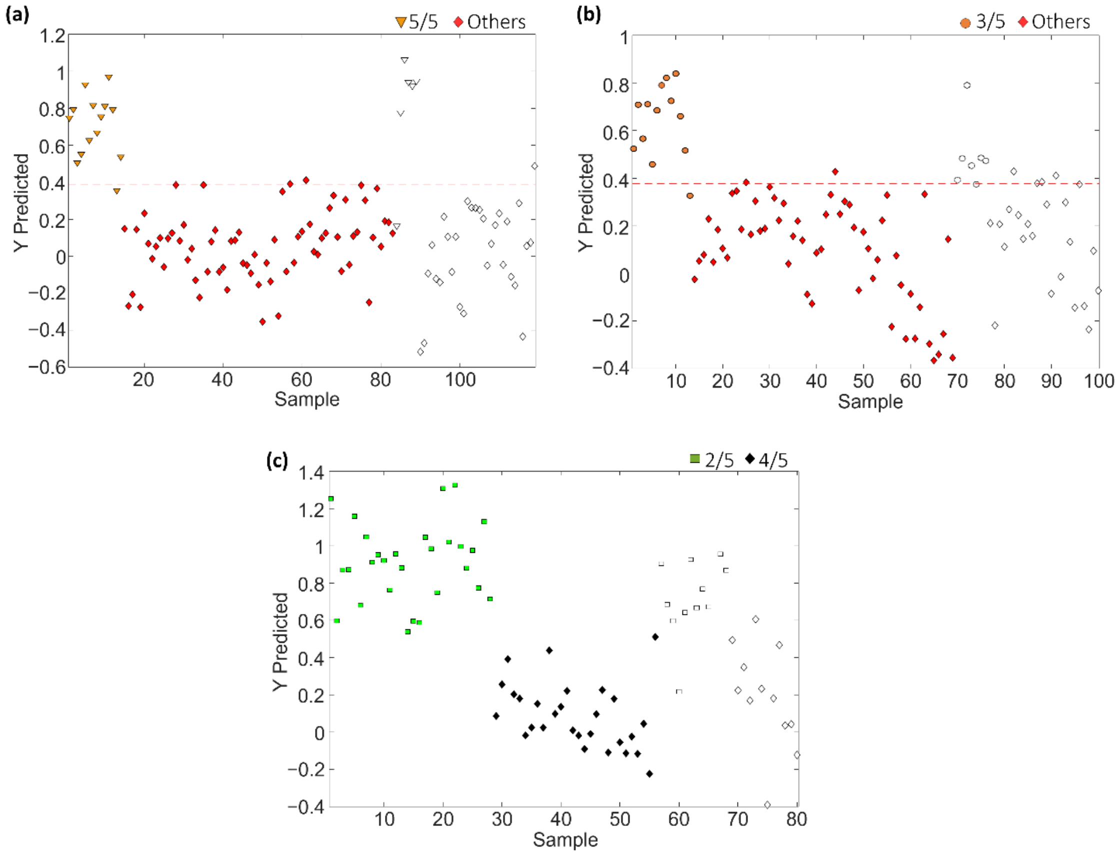

Figure S6. Paired PLS-DA plots of Y predicted *vs.* samples for set 2 within the tree classification framework: (a) 5/5 *vs.* Others, (b) 3/5 *vs.* Others and, (c) 2/5 *vs.* 4/5. Filled and empty symbols correspond to calibration and prediction sets, respectively. Red lines represent the threshold between classes.
